# Alcohol and Acetaldehyde in Public Health: From Marvel to Menace

**DOI:** 10.3390/ijerph7041285

**Published:** 2010-03-25

**Authors:** Rui Guo, Jun Ren

**Affiliations:** Center for Cardiovascular Research and Alternative Medicine, University of Wyoming, Laramie, College of Health Sciences, WY 82071, USA; E-Mail: rguo1@uwyo.edu

**Keywords:** alcohol, acetaldehyde, metabolism, human health

## Abstract

Alcohol abuse is a serious medical and social problem. Although light to moderate alcohol consumption is beneficial to cardiovascular health, heavy drinking often results in organ damage and social problems. In addition, genetic susceptibility to the effect of alcohol on cancer and coronary heart disease differs across the population. A number of mechanisms including direct the toxicity of ethanol, its metabolites [e.g., acetaldehyde and fatty acid ethyl esters (FAEEs)] and oxidative stress may mediate alcoholic complications. Acetaldehyde, the primary metabolic product of ethanol, is an important candidate toxin in developing alcoholic diseases. Meanwhile, free radicals produced during ethanol metabolism and FAEEs are also important triggers for alcoholic damages.

## 1. Introduction

The alcohol family is comprised of three different members namely methyl alcohol (methanol), isopropyl alcohol and ethyl alcohol (ethanol, EtOH or CH_3_CH_2_OH). The first two forms of alcohol are toxic and prohibited to consume. However, ethanol, or alcohol as commonly called, is an intoxicating ingredient in beer, wine and other forms of liquor. Over centuries, alcohol has become the most socially-accepted addictive drug worldwide. Alcohol beverages have long been known for their rather important role in social activities. Drinking alcoholic beverages is a common feature of social gatherings. Although light to moderate drinkers tend to display an overall better cardiovascular health and longevity compared with abstainers or heavy drinkers [1–4], long-term alcohol misuse or binge drinking can result in life-threatening health hazards both physically and mentally. Moreover, genetic susceptibility to alcohol-associated risk prevalence of cancer and coronary heart disease differs across the population. Therefore, it is recommended that moderate drinking must be individualized to reflect the potentially confounding effects of alcohol on several chronic diseases [5]. For example, individuals with a high risk of carcinogenesis should abstain from alcohol use [6]. Certain devastating chronic diseases such as heart disease [7–9], Alzheimer’s disease [10], stroke [11,12], liver disease [13–15], cancer [16–18], chronic respiratory disease [19,20], diabetes mellitus [21–23] and bone disease [24,25] may develop following chronic alcohol ingestion and contribute to the alcoholism-related high morbidity and mortality. In addition to chronic diseases, alcohol abuse may also trigger a cascade of acute health problems such as traffic accident-related injuries. Furthermore, social problem can also be a consequence of alcohol abuse including domestic violence, loss of work-place productivity, economic burden on society, crime and public disorders [26–29]. With the increased alcohol consumption in women and adolescences [30], alcohol-related social and health problems are attracting more and more attention.

To-date, a number of theories have been postulated for the pathogenesis of alcohol-induced complications including direct toxicity of ethanol and its metabolites [31], oxidative stress, accumulation of fatty acid ethyl esters [32] as well as modifications of lipoprotein and apolipoprotein particles [33]. In particular, acetaldehyde, the primary metabolic product of ethanol, is thought to be a candidate toxin and plays a pivotal role in priming and developing alcoholism [34]. Genetic polymorphism in alcohol dehydrogenase (ADH) [35] and aldehyde dehydrogenase (ALDH) [36,37], the two key enzymes responsible for ethanol/acetaldehyde metabolism, is involved in the susceptibility to alcoholism and alcohol-related organ damage and diseases. Ethanol elimination occurs through oxidation to acetaldehyde and acetate by way of ADH and ALDH, respectively. Different levels of blood acetaldehyde are shown in different genotypic verifications in the ADH or ALDH gene following alcohol intake [37], thus predisposing these individuals to alcohol damage, and the degrees of polymorphism differs depending on racial and ethnic groups [38].

## 2. Alcohol and Human Health

Since the beginning of last century, a number of studies have demonstrated that light to moderate alcohol consumption is associated with better cardiovascular health and longevity outcome compared with either abstainers or heavy drinkers [34,39]. One of the earliest scientific studies on the subject appeared in the *Journal of the American Medical Association* in 1904. In addition to reducing the risk of heart attacks, e.g., coronary heart disease (CHD), ischemic heart disease, atherosclerosis, angina pectoris [40–44], light to moderate drinking is also generally beneficial in minimizing the risk of stroke [45], peripheral artery disease [46], hypertension [35,47], liver disease [48], Alzheimer’s disease, Parkinson’s disease, diabetes [49–51], rheumatoid arthritis [52], bone fractures and osteoporosis [53,54], digestive ailments [55], stress and depression [56], renal cell carcinoma [57], pancreatic cancer [58], duodenal ulcer [59], macular degeneration [60], hearing loss [61], gallstones [62], poor physical condition in the elderly [63] and common cold [64]. Although the benefits and risks associated with light to moderate drinking have gained increasing attention in recent years from both researchers and the general public [39], no universal definition of moderate drinking has been established. The currently accepted definition for moderate drinking employs pure ethanol contained in “one drink” as a unit quantity to evaluate the amount consumed in a specific time period (e.g., USA and Canada, 12 or 14 g; Australia, 10 g; UK and Ireland, 8 g; Italy and Spain, 10 g; Denmark and France, 12 g; Japan, 20 g) [65]. There is also an indication to use 24 g ethanol, or two US standard drinks or less in a day, as the moderate alcohol intake [66].

Despite the beneficial effects of light to moderate alcohol intake, an ample of clinical and experimental evidence has demonstrated the concordant J or U-shaped associations between alcohol intake and a variety of adverse health outcomes [1]. Long-term alcohol misuse or heavy drinking not only failed to improve the health outcome but also enhanced the risk of various human diseases such as those mentioned previously. Binge drinking may cause detrimental damage to human organs including brain, liver, heart, lung, skeletal muscle and bones. For example, the brain may be affected resulting in confusion and memory loss [67–69]. The liver, the main site of ethanol oxidation, is extremely vulnerable to alcoholic damage [70,71], leading to cirrhosis, a severe form of liver disease and a major cause of death in the United States [72,73]. Excessive ethanol consumption also results in cardiovascular disease (the number one cause of death in the US), including ventricular dysfunction [74,75], dilated cardiomyopathy [74], ventricular arrhythmias [76], myocardial fibrosis [77] as well as enhanced risk of stroke and hypertension [78,79]. These morphological and functional defects of myocardium will eventually result in heart failure.

It should be emphasized that moderate drinking is recommended to be individualized to reflect the potentially competing or confounding effects of alcohol on certain chronic diseases [5]. It was indicated that moderate drinking had no beneficial effect on mortality in young adults (premenopausal women and men <40 years of age). Nonetheless, it is speculated that moderate drinking in young adulthood may dampen the risk of heart diseases later on in life. In certain populations, such as pregnant women, heavy drinkers and those on medication that may interact adversely with alcohol, the risk of alcohol consumption, even in the form of moderate ingestion, outweighs potential benefits [80]. Somewhat along this line, Sun and colleagues found that light to moderate alcohol use may provide the optimal benefit in older adults with poor health condition [81]. Evidence from the 2007 World Cancer Research Fund and American Institute for Cancer Research summary report recommended individuals with high risk of cancer are not recommended to drink alcoholic drinks despite the fact that modest amounts of alcoholic drinks are likely to reduce the risk of coronary heart disease [6]. Evidence from Allen and colleagues revealed that moderate alcohol use may particularly increase the risk of certain cancers such as breast and liver cancers while reducing the risk of some other cancers in women. Moreover, the alcohol-associated risk for upper aerodigestive tract cancer (oral cavity, esophagus, larynx and pharynx) was confined in active smokers, with little effect of alcohol use in never and past smokers [82]. Nonetheless, it is rather difficult to conclude whether the increased risk of cancer was due to alcohol intake or smoking since the two behaviors tend to be concurrent quite often. Although further work is still needed to fully consolidate the correlation between cancer prevalence and moderate alcohol intake, the American Cancer Society recommends limited alcohol use in both men (<2 drinks per day) and women (<1 drink per day). Taken together, whether moderate alcohol use plays a protective, unrelated or adverse role in human health is still controversial, depending heavily on age, gender and type of alcoholic beverage.

## 3. Mechanisms of Alcoholic Diseases

A number of mechanisms have been postulated for the pathogenesis of alcoholic injuries and diseases, including toxicity of ethanol and its metabolite acetaldehyde, the primary metabolic product of ethanol. In addition, oxidative stress, accumulation of fatty acid ethyl esters and modification of lipoprotein and apolipoprotein particles [33] also contribute to alcohol-associated complications. Although alcohol exposure is associated with multiple toxic effects on various organs through different mechanisms, two main categories are worth considering: acetaldehyde-related and non-acetaldehyde-related mechanisms.

### 3.1. Mechanism of Alcohol Metabolism

Ethanol metabolites and oxidative stress (through accumulation of reactive oxygen species—ROS) are thought to be the main causes of alcohol-induced organ damage. A majority of ethanol is metabolized in the cytoplasm of the liver by the enzyme ADH to produce acetaldehyde, which is then further metabolized to another less active byproduct, acetate, by ALDH [83]. The two enzymatic steps both require NAD as the hydrogen acceptor. The enzymes cytochrome P450 2E1 (CYP2E1) and catalase also break down alcohol to acetaldehyde. However, CYP2E1, the enzyme in the E subfamily of the second family P450s, becomes active only after a person has consumed large amount of alcohol. Under normal conditions, CYP2E1 accounts for less than 10% of ethanol metabolism. Catalase also metabolizes only a small fraction of alcohol without requiring NAD as a cofactor [83]. All these ways of metabolizing ethanol result in acetaldehyde, a primary metabolic product of alcohol. Acetaldehyde is a key generator of free radicals and a known carcinogen. Moreover, high levels of NADH in mitochondria can cause an increase in the number of superoxide (O_2_^−^) free radicals leading to the formation of hydroxyl radicals (OH^−^), lipid peroxidation and damage to mitochondria DNA [84]. High levels of free radicals diminish or impair the antioxidant homeostasis, leading to tissue damage. In addition, ethanol may induce up to a 10-fold up-regulation of CYP2E1 in the liver, which may be responsible for alcoholism-triggered oxidative damage [85–87]. Evidence has indicated that small amounts of alcohol may be removed via interaction with fatty acids to form fatty acid ethyl esters (FAEEs), the latter has been shown to contribute to damage to the heart, liver and pancreas [88,89].

### 3.2. Acetaldehyde-Related Mechanism in Alcohol-Induced Damages

Acetaldehyde, an organic chemical compound (CH_3_CHO or MeCHO), is an active metabolite that induces a range of toxic, pharmacological and behavioral responses. Although acetaldehyde is only short-lived prior to its breakdown into acetate, it possesses the ability to elicit overt cellular and tissue damage. The liver is often considered the primary site of oxidation [90], although other organs including the heart, pancreas, gastrointestinal tract and the brain, may also participate in the ethanol metabolism to form acetaldehyde [16,83,88,89]. Acetaldehyde causes mitochondrial dysfunction and in turn compromises acetaldehyde metabolism to result in the accumulation of acetaldehyde, leading to a vicious cycle. Acetaldehyde may also react with amino, hydroxyl, and sulfhydryl groups to interfere with or modify the structure and function of macromolecules in the body, such as proteins and enzymes [89,91].

Accumulating evidence suggested that acetaldehyde plays a key role in the pathogenesis of alcoholic cardiomyopathy [92–97]. In particular, acetaldehyde has been shown to lead cardiac hypertrophy or dilated cardiomyopathy associated with significant increase in the hypertrophic marker skeletal actin and ANF [97]. Data from our laboratory have shown that acetaldehyde compromises myocardial excitation-contraction coupling, sarco (endo) plasmic reticulum [42] Ca^2+^ release and cardiac contractile function [34,75,98,99]. The mechanism underlying acetaldehyde-induced myocardial depression may be due, in part, to either reduced Ca^2+^ entry through voltage-dependent Ca^2+^ channels and/or depression of sarcoplasmic reticular Ca^2+^ release [75]. Our previous study showed that alcohol intake significantly reduced expression of the intracellular Ca^2+^ cycling proteins SERCA2a, Na^+^-Ca^2+^ exchanger and phospholamban in cardiomyocytes without overt change in the SERCA2a-to-phospholamban ratio [100]. Although the precise mechanism behind alcohol-induced change in the intracellular Ca^2+^ regulatory proteins is not fully clear, acetaldehyde is believed to play a role. Acetaldehyde was recently suggested to function as a ryanodine receptor activator to leading to disturbed cardiac contractile function [101] and elevated intracellular Ca^2+^ levels [102]. Acetaldehyde stimulates the release of signaling molecules (epinephrine, norepinephrine, histamine and bradykinin) and leads to the cardiovascular symptoms of the alcohol sensitivity reaction such as vasodilation and facial flushing. It also associated with abnormal heart beat and blood pressure [103]. As the major metabolite of ethanol, acetaldehyde production results directly in the formation of free radicals through aldehyde oxidase and xanthine oxidase-associated oxidation and indirectly in decreased antioxidant defenses (e.g., GSH levels) [34,104,105], resulting in oxidative stress. Acetaldehyde can also induce apoptosis via activation of stress signaling such as c-Jun phosphorylation [34,106,107]. This is supported by our experimental findings of elevated TUNEL-positive apoptotic cells in ADH murine hearts following ethanol challenge.

In addition to direct cytotoxicity, acetaldehyde-associated organ damage may also be mediated through inflammatory cytokines (e.g., tumor necrosis factor and the interferons), as well as the binding capability to certain proteins [108–110]. In addition to direct organ damage, acetaldehyde may also be responsible for certain behavioral and physiological effects previously attributed to alcohol. For example, when acetaldehyde is administered to lab animals, it leads to uncoordination, memory impairment, and sleepiness, effects often associated with alcohol ingestion [103]. Moreover, the acetaldehyde-DNA binding has been considered to promote carcinogenesis in alcohol-dependent individuals [111]. Similarly, formation of crotonaldehyde [66] from acetaldehyde is also known as a potentially carcinogenic pollutant [112]. Paradoxically, acetaldehyde may also contribute to the beneficial effect following light to moderate alcohol intake. It was reported that attachment of acetaldehyde to a model Amadori product produces a chemically stabilized complex that cannot rearrange and progress to formation of advanced glycation endproducts, or AGEs [113]. Amadori products typically arise from the nonenzymatic addition of sugars to protein amino groups and are the precursors to the irreversibly bound, crosslinking moieties of AGEs, which are detrimental to health. Therefore, acetaldehyde-induced protein adduct may contribute to the beneficial effect of light to moderate alcohol intake, or the so-called “French paradox” by inhibiting advanced glycation.

### 3.3. Non-Acetaldehyde-Related Mechanism in Alcohol-Induced Damages

Recent evidence also indicates the contribution of acetaldehyde-independent mechanisms to the pathogenesis of alcoholic diseases. For example, ethanol may elicit direct toxic effects on the cardiovascular system or alter neurohumoral and/or hormonal regulation of cardiac function [78]. Certain metabolic product of ethanol such as fatty acid ethyl esters (FAEEs) may also interfere with the physiological function of the heart independently of acetaldehyde. The formation of FAEEs in the heart is an example of a non-oxidative metabolism of alcohol, and is distinguished from the oxidative metabolism of alcohol in the liver. FAEEs may prove to be the first link between the ingestion of alcohol and the development of alcohol-induced heart muscle disease. Although the amount of fatty acids in heart muscle is small, following consumption of alcohol, FAEEs concentration in the human myocardium can accumulate 115,000-fold higher than in the normal heart muscle [114,115]. Accumulation of fatty acid ethyl esters is capable of reducing the respiratory control ratio index of coupling of oxidative phosphorylation and maximal rate of oxygen consumption, and accounts for impaired mitochondrial function and inefficient energy production associated with toxic effects of ethanol on the heart [115]. Data from our group also suggested that acetaldehyde-induced cardiac mechanical dysfunction may be ablated by folate or thiamine supplementation [116,117], suggesting a possible interaction between acetaldehyde-induced cardiac toxicity and nutritional status. This is somewhat consistent with the favorable response of patients with alcoholic cardiomyopathy to thiamine and nutrition treatment [118].

Meanwhile, ethanol metabolism also produces stable and unstable protein adducts. For example, acetaldehyde binds to some proteins and becomes a Schiff base, thus forming protein-acetaldehyde adducts. Furthermore, the lipid peroxide-derived aldehydes such as malondialdehyde (MDA) and 4-hydroxynonenal may eventually form stable hybrid adducts by potential protein modifications. These multiple adducts were shown to have a cadre of inverse effects on the cells of the immune system and involved in the development of alcoholic organ disease, including liver, heart and brain [110,119].

### 3.4. Genetic Polymorphisms of Alcohol Metabolizing Enzymes

Alcoholism has been considered to be associated with different genetic factors in alcohol metabolism in different ethnic groups. Studies have identified numerous functional polymorphisms in genes encoding enzymes for ethanol metabolism. According to recent studies, the human ALDH gene superfamily comprises 19 genes encoding enzymes critical for NAD (P)^+^—dependent oxidation of endogenous and exogenous aldehydes [120]. Meanwhile, there are at least seven genes in the ADH family [121]. *ADH2*, *ADH3*, and *ALDH2* are thought to be the pivotal genetic determinants in ethanol metabolism and alcoholism in humans.

Within the nine major members of ALDH families, mitochondrial ALDH2 has a rather unique role in aldehyde detoxification [122]. Deficiency in ALDH2 expression and/or activity is responsible for facial flushing and other vasomotor symptoms following alcohol ingestion. In addition, findings from Kawamoto’s group indicated that deficiency in ALDH2 enzymatic activity inhibits acetate formation via acetaldehyde [123]. Prevalence of the ALDH2*1 allele is associated with alcoholism. Deficiency in ALDH2 due to point mutation in the active ALDH2*1 gene, significantly alters blood acetaldehyde levels and vulnerability for alcoholism [124]. However, ALDH2*2, which is dominant over ALDH2*1, encodes a glutamate to lysine substitution at residue 487 in the mature enzyme, resulting in a loss of enzymatic activity [122]. In addition, individuals carrying the ADH2*2 allele display slightly facilitated alcohol metabolism due to higher enzyme activity compared with ADH2 *1 encoding populations [125].

Research reported that the allele frequencies of the genes ADH2*2 and ALDH2*2 were lower in Northwest Coast Amerindians, Africans, Europeans and Australian Aborigines than South America Indians and Asians including Chinese, Japanese and Koreans [125–127]. The ALDH2*2 allele encodes an inactive subunit of ALDH2, which consists of four subunits, and ALDH2 shows lack of activity when even one inactive subunit protein is included [128]. Therefore, Asians always cannot drink a great amount of alcohol as compared with Caucasians.

Some studies also indicated that those who carry ALDH2*2 alleles were strikingly responsive to a small amount of alcohol. Large accumulation of acetaldehyde in the blood of these people produces a pattern of uncomfortable effects such as increasing skin temperature and facial flushing, dropping blood pressure, nausea, headache, palpitations and bronchoconstriction [103]. Of certain significance, some researchers suggested that acetaldehyde causing these symptoms may provide a protective role against heavy drinking intake, otherwise, alcoholism or worse outcome [36]. It has been suggested that the mutant ALDH2 gene of ALDH2*2/2 may protect against development of alcohol dependence and alcohol-related disease [129]. Nonetheless, this sort of epidemiological study fails to offer direct evidence regarding the role of acetaldehyde on cardiac function due to intolerance to alcohol among these individuals with genetic polymorphisms [124]. Our recent observation from animal study provided some convincing evidence regarding the role of ALDH2 in ethanol-induced cardiac toxicity. Overexpression of ALDH2 was found to be cardioprotective against acute ethanol-induced cardiac toxicity, possibly through inhibition of protein phosphatases. Our data further revealed that enhanced activation of Akt and AMPK, and subsequently, inhibition of Foxo3, apoptosis, and mitochondrial dysfunction may play a pivotal role in ALDH2 overexpression-induced cardioprotection against ethanol toxicity [130].

Alcoholism is a multifactorial disease including a complex mode of hereditary, psychological and social factors. More in depth genetic association studies is warranted to further identify and consolidate the genetic risk factors for alcoholism. Furthermore, according to the differing polymorphisms among racial populations, effective measures can be appropriately taken for the studies.

## 4. Alcohol-induced Social Problems and Effective Ways to Reduce Alcohol Abuse

Alcohol abuse and addiction are not only an individual problem but also a social problem. Alcohol abuse is closely associated with the society such as due to car accidents, social violence, broken homes, productivity losses, child abuse and any other crimes. Underage drinking is another serious public health concern in children and adolescents [131]. Researchers have suggested that people with a psychiatric condition called antisocial personality disorder may be particularly susceptible to alcohol-related aggression [27]. Alcohol may also affect female reproductive function at several stages of life. It has been shown to elicit a detrimental effect on puberty, to disrupt normal menstrual cycle and to alter hormonal levels in postmenopausal women. In addition, alcohol abuse also increases the economic burden on society [26,29]. Certain strategies were reported to reduce alcohol abuse, such as increased taxes and prices of alcoholic beverages, raising the Minimum Legal Drinking Age, setting maximum blood alcohol concentration (BAC) limits for drivers under 21, making warning labels on containers of alcoholic beverages, as well as community and educational interventions, e.g., alcohol misuse prevention study (AMPS) and drug abuse resistance education (DARE).

## 5. Conclusion

Given that alcohol drinking-induced effects may exhibit great individual variation, it is rather cumbersome to figure out where the line is between social drinking and problem drinking. Chronic alcohol ingestion or binge drinking may trigger detrimental bodily damage, which is heavily influenced by many interconnected factors such as races, genetics, environment and the emotional health. It is generally accepted that light to moderate alcohol consumption is beneficial to reduce the risk of some human diseases, although it may increase the risk prevalence for certain cancers especially in women. In addition, heavy drinking has been consistently found harmful and dangerous (with lethal damage such as cancer, heart and liver disease). Moreover, the social consequences of alcohol abuse can be just as devastating. In particular, the emerging trend of more harmful and risky drinking among young people and among women may have a severe health outcome [132]. It is essential to understand the drinking problems, the definition of moderate drinking, alcoholism and alcohol abuse. In order to minimize the harmful sequelae of alcohol use, it is also important to raise awareness in individual’s genetic trait in alcohol metabolizing enzymes ADH and ALDH, and then to apply corresponding and necessary measures (for example delay the drinking onset age) [133] to minimize the alcoholic injury.
